# Investigation on the Properties and on the Photo-Oxidation Behaviour of Polypropylene/Fumed Silica Nanocomposites

**DOI:** 10.3390/polym13162673

**Published:** 2021-08-10

**Authors:** Vincenzo Titone, Maria Chiara Mistretta, Luigi Botta, Francesco Paolo La Mantia

**Affiliations:** 1 Department of Engineering, University of Palermo, Viale delle Scienze, 90128 Palermo, Italy; vincenzo.titone@unipa.it (V.T.); mariachiara.mistretta@unipa.it (M.C.M.); luigi.botta@unipa.it (L.B.); 2 INSTM Consortium for Materials Science and Technology, Via Giusti 9, 50125 Florence, Italy

**Keywords:** nanocomposites, polypropylene, silica, rheology, mechanical properties, photo-oxidation

## Abstract

This work investigates the effects of very small amounts of fumed silica on the morphology and on the rheological and mechanical behaviour of polypropylene nanocomposites and on their photo-oxidation behaviour. Polypropylene nanocomposites were prepared using a twin-screw corotating extruder with 0, 1 and 2 wt/wt% of SiO_2_. Morphological, mechanical, thermomechanical and rheological properties were examined. It was found that the viscosity of the matrix is reduced by the presence of the silica nanoparticles, suggesting a poor adhesion between the two phases and probably some lubricating effect. On the contrary, the mechanical and, in particular, the thermomechanical properties of the matrix are considerably improved by the presence of the silica. In particular, elastic modulus and tensile strength increases remarkably, and this effect becomes more and more remarkable with an increasing temperature. As for the photo-oxidation behaviour, the presence of silica improves the photostability of the polypropylene matrix. This effect has been attributed to both the barrier to the oxygen and to the absorbance of the UV radiation from the silica nanoparticles. Finally, no significant effect of the silica nanoparticles has been put in evidence on the crystallisation behaviour of the polypropylene. As for the effect of the silica content, the difference in the properties of the two nanocomposites is relatively small and all the measured properties depend much less than linearly with its amount. This has been correlated with the reaggregation of the nanoparticles that, having a larger size, decrease the contact area between the matrix and the filler.

## 1. Introduction

Nowadays, polymer nanocomposites have attracted the attention of many groups in both the scientific community and industry. Several studies, indeed, have shown that incorporating very small amounts of nanoparticles into thermoplastic materials can improve properties, such as mechanical properties, [1,2] thermal, [3,4] barrier, [5,6] and dielectric [7].

Polypropylene is a thermoplastic material used widely in industrial applications because of its excellent processing ability, low cost and, especially, recyclability. Although it can be produced with different molecular chain structures under controlled conditions (stereospecific), the most commercially attractive form is still the isotactic form, which is a semicrystalline polymer characterised by high tensile strength, low density, low thermal and abrasion resistance. Moreover, the isotactic form also provides good mechanical strength, particularly flexural strength.

Over the years, several nano-reinforcing materials have been introduced in polypropylene, such as calcium carbonate [8,9,10], nano-clay [11,12], graphene [13,14,15,16] and nano-silica as well [17,18].

The reported results indicate that due to its reinforcing effect, the mechanical properties of polypropylene can be improved by adding silica nanoparticles. Nevertheless, due to the non-polar nature of polypropylene and the large surface area of the polar nano-silica particles, it is difficult to achieve a good dispersion of the silica nanoparticles in the polypropylene matrix. For this reason, surface-treated nano-silica [19,20,21] or compatibilizers [22,23] have been used to improve the dispersion and the adhesion of nano-silica in polypropylene. In fact, the use of compatibilizers such as maleic anhydride improves the adhesion between polypropylene and silica, and thus, due to the reinforcing effect of well-dispersed silica particles, it is possible to obtain high ductility and increase the impact strength of nanocomposites. To the best of our knowledge, only one paper considers the photostability of nanocomposite with silica [24]. In this paper, the presence of silica improves the photostability of the nanocomposite.

We focused this study on the preparation and the characterisation of the morphological, mechanical, thermomechanical and rheological properties of polypropylene/silica nanocomposites. Moreover, the photo-oxidation behaviour has been investigated. Low filler loadings (1 and 2% by weight) were used. The presence of these small amounts of silica induces a remarkable increase in the rigidity of the polypropylene matrix and, in particular, of the thermomechanical resistance of the matrix. By adding only 1 wt/wt% of silica improves the temperature at which the modulus starts to decrease by about 30 °C. With an increasing silica content, the improvement is less than linear. This behaviour has been interpreted in terms of reaggregation of the nanoparticles with an increasing content.

Moreover, in the molten state, the presence of the silica decreases the viscosity of the matrix due to the poor adhesion. This is, of course, an advantage in the injection moulding process, where low viscosity is desired. Finally, the presence of the silica improves the photostability of the polypropylene matrix. This effect has been interpreted considering the barrier to the oxygen exerted by the nanoparticles of silica and the absorbance of the UV radiation by the silica nanoparticles. However, the protection is not proportional to the silica content, and this has been interpreted on the basis of the different dimensions of the nanoparticles with the silica content and on the consequent surface exposed to the oxygen.

## 2. Materials and Methods

### 2.1. Material and Processing

Polypropylene Moplen^®^ X30G produced by LyondellBasell (LyondellBasell, Ferrara, Italy) with a melt flow index (M.F.I.) of 8 g/10 min (230 °C/2.16 Kg) and density 0.90 g/cm^3^ was used for nanocomposite preparation. The filler, a fumed silica (Aerosil^®^ R812, Evonik Industries, Essen, Germany), with a content of 99.8% SiO_2_, tamped density of approximately 60 g/l and specific surface area of 230–290 m^2^/g was supplied by Evonik Industries (Evonik Industries, Essen, Germany).

Polypropylene (PP) and polypropylene nanocomposites (filler content 1 (PP1) and 2 (PP2) wt/wt%) were extruded using a coronating twin-screw extruder OMC (OMC, Saronno, Italy) with a screw diameter of 19 mm and length to diameter ratio of 35 mm. The temperature profile for polypropylene and nanocomposites was 130–140–150–160–170–180–190 °C (die), with screw speed set at 220 rpm and the gravimetric feeder set at 12 rpm. Before use, fumed silica was dried at 120 °C under vacuum for at least 12 h to remove any traces of moisture.

The specimens for the mechanical and rheological characterisation were prepared using compression moulding in a Carver (Carver, Wabash, IN, USA) laboratory hydraulic press at the temperature of 190 °C under a mould pressure of 300 psi for about 3 min.

### 2.2. Photo-Oxidation

In order to evaluate UV-B resistance, an accelerated aging test was carried out using a UV weathering chamber (Q-Labs Corp., Westlake, OH, USA) equipped with eight UVB-313 lamps (according to ASTM G53-96) and operated under the cycle conditions of 8 h UV exposure at 70 °C, followed by 4 h of condensation at 50 °C.

### 2.3. Characterisation

Tensile strength (TS) and elongation at break (EB) were determined using an Instron (Instron, High Wycombe, UK) universal testing machine model 3365 at a crosshead speed of 20 mm/min, whereas elastic modulus was measured at a speed of 1 mm/min. All the results represent an average value of minimum 8 tests. The tensile strength specimens were rectangular sheets according to ASTM D638-14 [25] (length—90 mm, width—10 mm, thickness—≈0.3 mm).

The rheological characterisation was performed at 190 °C using both an ARES G2 (TA Instruments, New Castle, DE, USA) plate–plate rotational and a Rheologic 1000 capillary viscometer (CEAST, Torino, Italy). The measurements in the rotational rheometer were made at 190 °C in an angular frequency range from 0.1 to 100 rad/s, using 25-millimetre diameter parallel plates. The capillary viscometer has a die with D = 1 mm and the length to diameter ratio was 40. Due to the large length-to-diameter ratio, Bagley correction was not applied, whereas the Rabinowitsch correction was applied throughout.

Thermal analysis was performed using differential scanning calorimetry (DSC) using a DSC-60 apparatus of Shimadzu (Shimadzu, Kyoto, Japan) under nitrogen gas atmosphere. The amount of sample placed in the DSC aluminium pans was about 8 ± 2 mg, while the heating rate was from 10 °C/min to 200 °C/min.

The degree of crystallinity (X_C_) was evaluated from the melting enthalpy results (ΔH_m_) of each sample using the following Equation (1) [26]:

(1) $$X_{C},\%=\left(\frac{{\Delta H}_{m}}{{\Delta H}_{100}\times W_{p}}\right)*100$$

where $\Delta H_{m}$ is the enthalpy of fusion, $\Delta H_{100}$ is enthalpy of fusion for 100% crystalline polymer and $W_{p}$ is the weight fraction of polymer. For polypropylene, $\Delta H_{100}$ = 207 J/g [27]. 

Dynamic mechanical thermal analysis was performed using a Metravib DMA 50 (Metravib, Limonest, France). The measurements were carried out at a constant frequency of ω = 1 Hz, a temperature range of 20 to 90 °C and heating 3 °C/min. Analyses were carried out on samples cut to size of 10× 30× ≈ 0.6 mm before being mounted in the DMTA apparatus.

Morphology was observed through scanning electron microscopy (SEM). SEM micrographs were obtained on samples fractured in liquid nitrogen and gold-sputtered (in order to make them conductive), using a Quanta 200 F (FEI Co., Hillsboro, OR, USA) scanning electron microscope. The SEM images with 20.000× amplification were processed with ImageJ.

FTIR spectra in ATR mode were obtained by using a Spectrum One spectrometer (Perkin-Elmer, Norwalk, CT, USA), equipped with integrated Spectrum One software. The spectra were obtained through 16 scans with a 4 cm^−1^ resolution. Measurements were obtained from the average of triplicate samples. 

UV–vis absorption spectra of the samples were measured with the Specord 252 spectrometer (Analytik Jena, Jena, Germany) in the range 190–500 nm.

## 3. Results and Discussion

### 3.1. Characterisation of Polypropylene Nanocomposites

**Figure 1:**
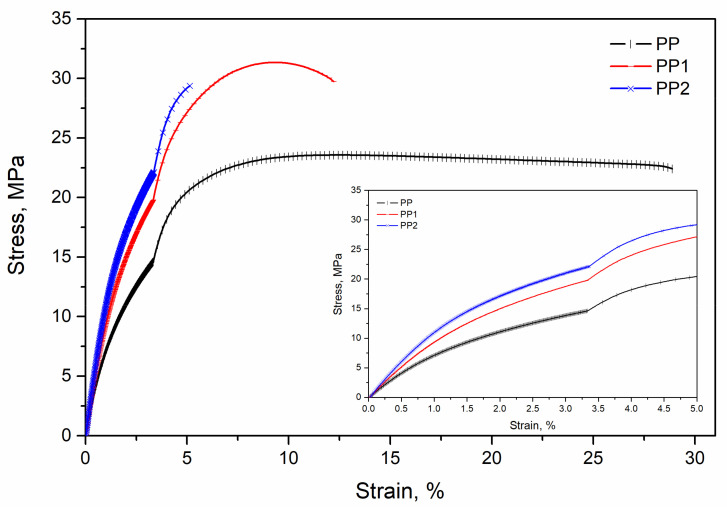
Stress–strain curve of polypropylene and polypropylene nanocomposites.

In Figure 1, the typical stress–strain curves of the three samples are reported. The discontinuity of the curves is due to the change of strain rate. The presence of the nanoparticles makes the nanocomposites samples more rigid and less deformable. Indeed, the nanocomposites show higher values of the elastic modulus and of the tensile strength, but lower values of the elongation at break.

**Figure 2:**
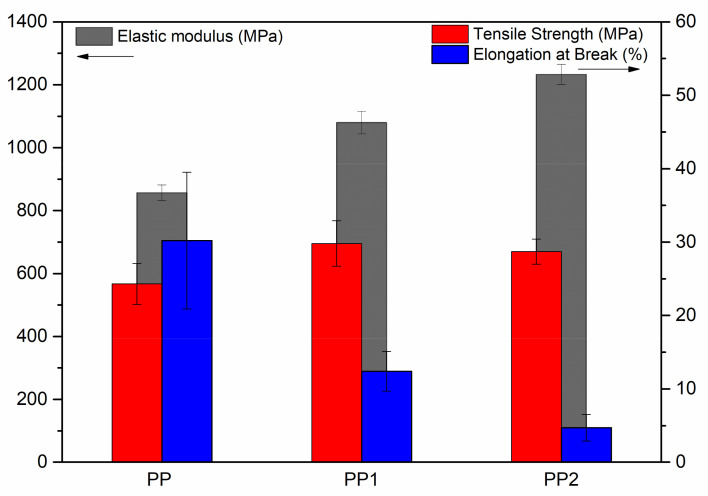
Histogram of elastic modulus, tensile strength and deformation at break of polypropylene and polypropylene nanocomposites.

In Figure 2, the results of the mechanical tests performed on neat polypropylene and polypropylene nanocomposites are reported.

The average elastic modulus, tensile strength and elongation at break values of neat polypropylene are about 857 MPa, 24.3 MPa and 30.2%, respectively. As observed in the stress–strain curves, in the case of polypropylene nanocomposites, the addition of very small amounts of silica nanoparticles causes an increase in the elastic modulus and in the tensile strength values with respect to those of the polymer matrix. The elastic modulus, indeed, increases rapidly from 857 to 1233 MPa for the nanocomposite with 2% of silica. The highest value of the tensile at strength is shown by the PP1 sample and not from the more filled PP2 sample. This can be attributed to the lower deformability of this last sample that causes a premature rupture of the specimen. Finally, the elongation at break decreases continuously with an increasing silica content. Indeed, the average value of 5% shown by the PP2 sample is much lower compared to the value of 30% of the matrix. The improvement of the mechanical properties is similar for the two samples of nanocomposites and then the dependence on the content of silica is relatively small.

**Figure 3:**
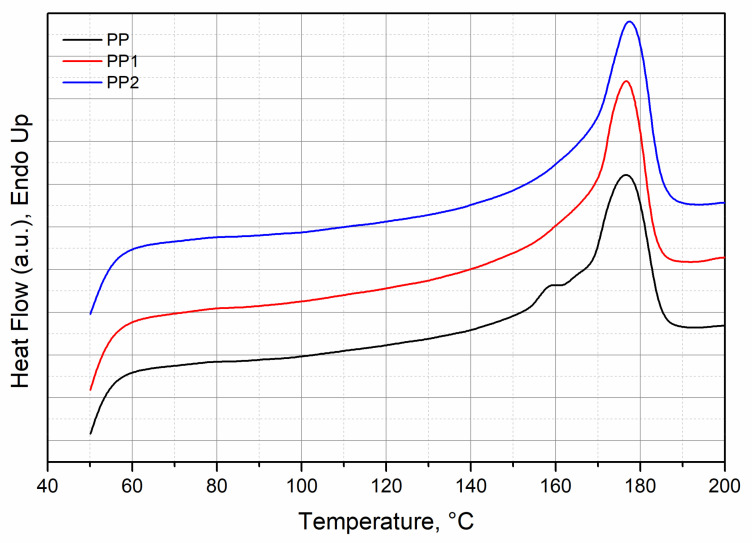
Thermograms recorded during the second heating scan of polypropylene and polypropylene nanocomposites.

Figure 3 shows the thermograms of all three samples.

As it can be observed from this figure, all the samples show a melting peak at about the same temperature for all the samples (see Table 1), although a very slight increase is observed in the presence of silica, whatever its content. The same comments can be made for the melting enthalpy and then for the degree of crystallisation, see Table 1.

**Figure 4:**
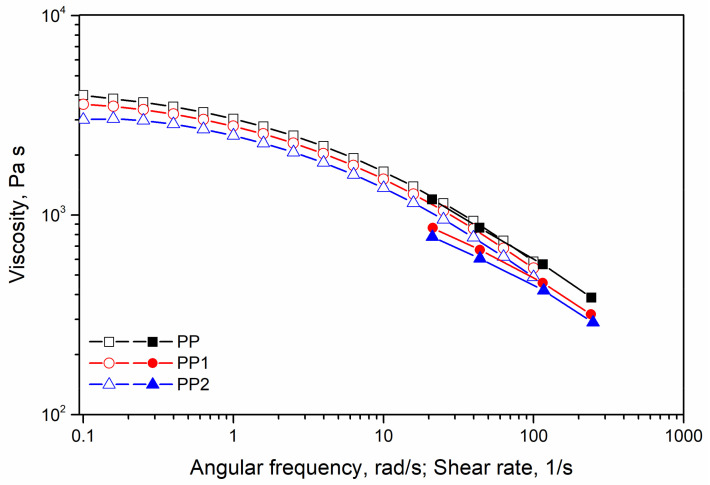
Flow curves of polypropylene and polypropylene nanocomposites. Date taken from rotational rheometer (open symbols) and capillary viscometer (closed symbols).

In Figure 4, the flow curves of polypropylene and polypropylene nanocomposites are reported.

Firstly, it can be observed that only the polypropylene matrix obeys the Cox–Merz rule and then, the superposition between the complex viscosity curve measured in the rotational rheometer and the flow curve measured in the capillary viscometer is observed. The polypropylene nanocomposites do not follow the Cox–Merz rule, and, indeed, the two curves do not superimpose, being the capillary flow curves at high shear rates lower than that of the complex viscosity. This result has already been described for heterogeneous, multiphase materials, such as polymer blends and polymer-based nanocomposites [28,29,30,31]. Surprisingly, the flow curves of the two nanocomposites show a viscosity lower than that of the neat matrix. This behaviour has, however [29], been observed and attributed to a slippage between the polymer chains and the filler particles. Of course, this means that the adhesion between the two phases is very poor in the molten state.

**Figure 5:**
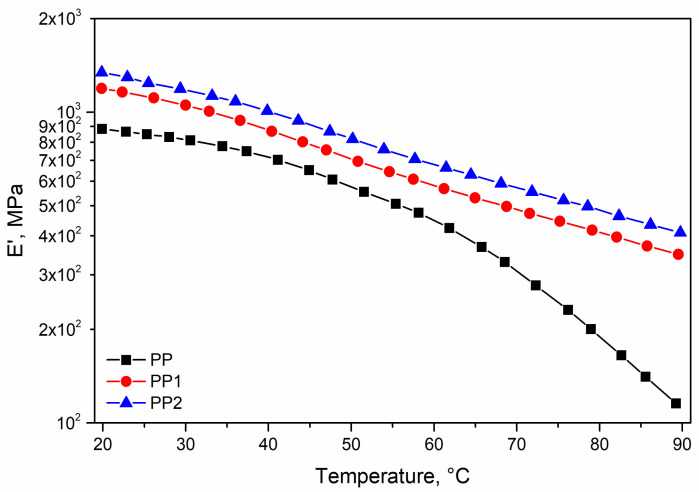
Storage modulus (E′) of polypropylene and polypropylene nanocomposites.

**Figure 6:**
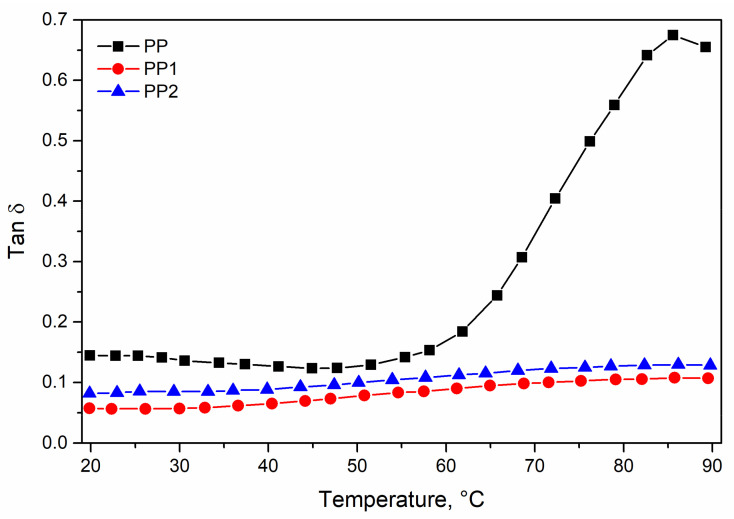
Loss factor (tan δ) of polypropylene and polypropylene nanocomposites.

In Figure 5 and Figure 6 the storage modulus, E′, and loss factor, tan δ, of the investigated polymer systems are reported as a function of the temperature. The effect of the presence of very low contents of silica in the range of the low temperature is slightly larger than that measured in tensile test, but the effect of the silica becomes very impressive with the increasing temperature.

Indeed, the E′ curve of the two nanocomposites is only slightly dependent on the temperature, while the E′ curve of the matrix falls down dramatically above 60 °C. At the temperature of 90 °C, the E′ value of PP2 is about the same of the matrix at the temperature of 60 °C. At the same temperature, the value of E’ is about six times that of the matrix. The improvement of the thermomechanical resistance is slightly better for the sample PP2.

On the corresponding tan δ curves, it can be observed that in the investigated temperature range, the two nanocomposites show an almost flat trend, while a dramatic increase in the tan δ curve is observed for the neat matrix responsible for the dramatic decay of the storage modulus.

**Figure 7:**
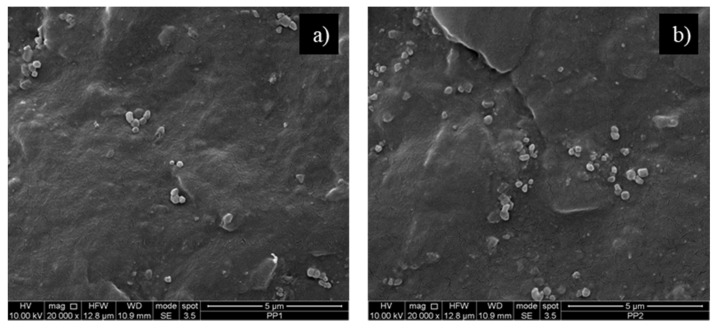
Scanning electron microscopy micrographs of the two polypropylene nanocomposites: (**a**) PP1 and (**b**) PP2.

In order to study the dispersion and distribution of the nanoparticles of silica, the fracture surface of the two nanocomposites was analysed using scanning electron microscopy, see Figure 7.

From the two micrographs, it is possible to observe a scarce adhesion between the matrix and the silica and a quite good dispersion, especially for the sample with 1% of silica. Moreover, the dimensions of the nanoparticles in the two samples are quite different and the sample PP2 shows larger nanoparticles than that of the sample PP1.

**Figure 8:**
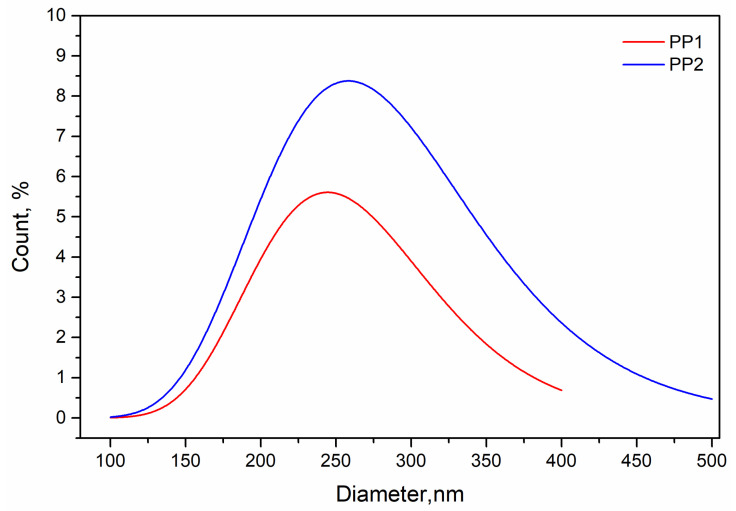
Distribution of the equivalent diameters of the two polypropylene nanocomposites.

In Figure 8**,** the curves of distribution of the equivalent diameters of the nanoparticle of the two nanocomposites are reported.

The PP1 sample shows nanoparticle sizes in the range 100–400 nm, while the sample PP2 shows sizes in the range 100–500 nm. This means, of course, that increasing the amount of silica nanoparticles, the dispersion is worse, and the nanoparticles tend to reaggregate. By evaluating these curves, an average weighted diameter can be calculated as follows:

(2) $$\bar{D}=\overset{n}{\underset{i=1}{\sum }}(D_{i}\times n_{i})/n_{i}$$

The values calculated for the two nanocomposites are about 255 and 285 for PP1 and PP2, respectively.

As observed before, the improvement of the mechanical properties is relatively low, going from 1 to 2% of nanofiller; nevertheless, this is a doubling of the content of the silica. The mechanical properties of a composite depend on the morphology and properties of the two phases and on the adhesion between matrix and filler. Indeed, the stress is transmitted from the matrix to the reinforcing filler through the contact area between the two phases. The adhesion, of course, depends on the chemical nature of the two components, but the value of the contact area depends on the particle size. The smaller the particle size is, the larger the contact area matrix-filler is, and this improves the transmission of the stress from the matrix to the filler.

The contact area can be evaluated by considering the surface area of each nanoparticle or aggregate by the number of particles [32]. In particular, the contact area of each particle is proportional to its square diameter, as follows:

(3) $$A_{i}\propto D_{i}^{2}$$

and the number of particles is as follows:

(4) $$N_{i}=Vt_{i}/V_{i}$$

where $Vt_{i}$ is the volume of the silica in the sample and $V_{i}$ is the volume of each single silica particle.

The total contact area of each sample is then as follows:

(5) $$A_{i}t=A_{i}N_{i}=\frac{A_{i}Vt_{i}}{V_{i}}\propto \frac{D_{i}^{2}Vt_{i}}{D_{i}^{3}}\propto \frac{Vt_{i}}{D_{i}}$$

The ratio between the contact area of PP2 with respect to PP1 is as follows:

(6) $$A_{2}t/A_{1}t=\frac{Vt_{2}D_{1}}{Vt_{1}D_{2}}$$

where $A_{2}t$ and $A_{1}t$ are the contact area of the samples PP2 and PP1, respectively.

By using the average equivalent diameters reported above, $A_{2}t/A_{1}t$ ≃ 1.6 and then the contact area of PP2 is twice that of PP1. This is in agreement with the mechanical results that increase less than linearly with the silica content.

### 3.2. Photo-Oxidation

**Figure 9:**
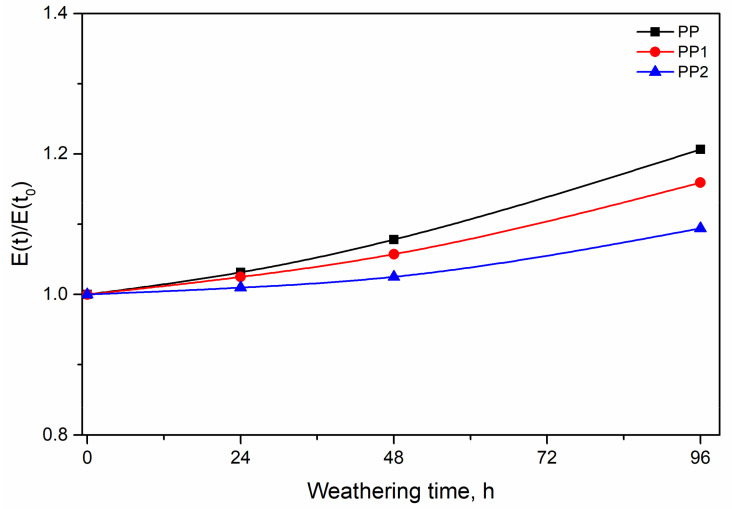
Relative elastic modulus as a function of the weathering time.

The relative values of the elastic modulus, E, is reported in Figure 9 as a function of the weathering time. The relative values have been calculated as the ratio between the modulus at a given weathering time divided by the modulus of the unexposed sample. The elastic modulus increases with the weathering time for all the samples. This behaviour has been already observed in other polymer systems [26,32,33,34] and has been attributed to the decrease in the molecular weight due to the photo-oxidation and the consequent increase in the degree of crystallinity (see Table 2). The crystallinity degree increases because the lower molecular weight provokes a larger crystallisation.

Although the crystallinity of the nanocomposites is larger, the increase in the crystallinity of the neat PP is larger, as put in evidence by the dimensionless values reported in the same table, giving rise to a larger increase in the modulus.

**Figure 10:**
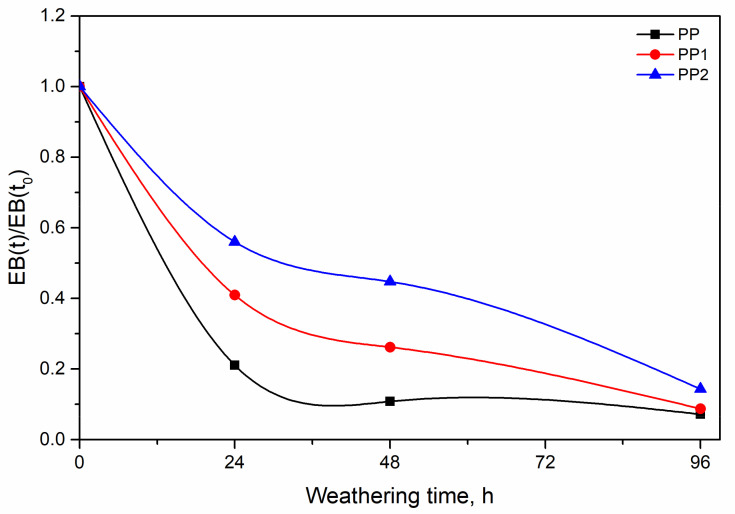
Relative elongation at break as a function of weathering time.

**Figure 11:**
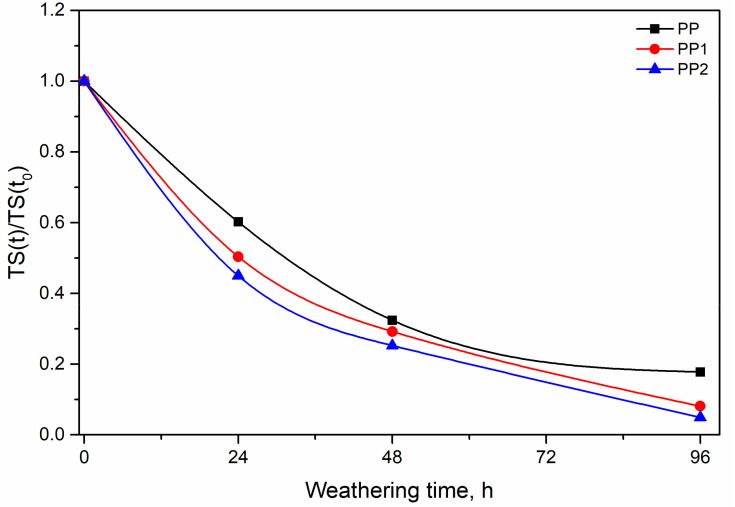
Relative tensile strength as a function of the weathering time.

The two nanocomposite samples show a lower increase and the PP2 sample shows the lowest increase in the modulus. This behaviour suggests a protective effect of the silica nanoparticles Indeed, a lower increase in the modulus should imply, according to our previous hypothesis, a lower level of degradation. The same protective effect is shown by the lower decay of the curves of TS and EB with exposure time, see Figure 10 and Figure 11.

**Figure 12:**
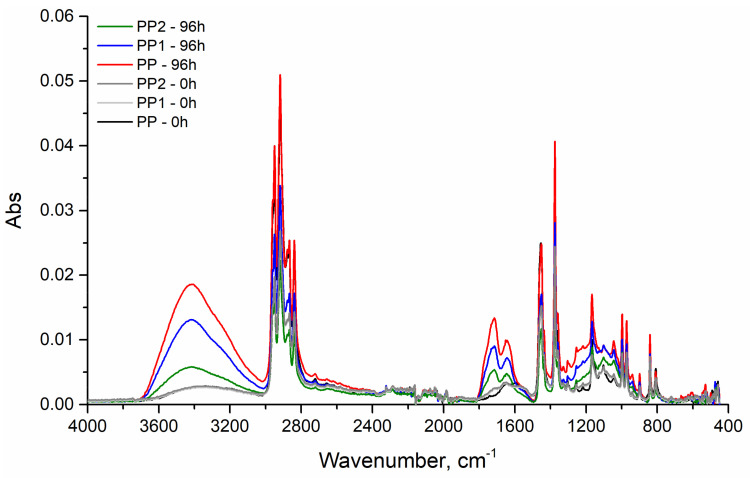
ATR spectra of the polypropylene and polypropylene nanocomposites after 96 h of exposure.

The FTIR spectra, Figure 12, confirm this hypothesis. Indeed, the bands of the oxygenated groups (1800–1650 cm^−1^) grow dramatically for the pure PP and very low for the nanocomposite with the largest amount of silica.

**Figure 13:**
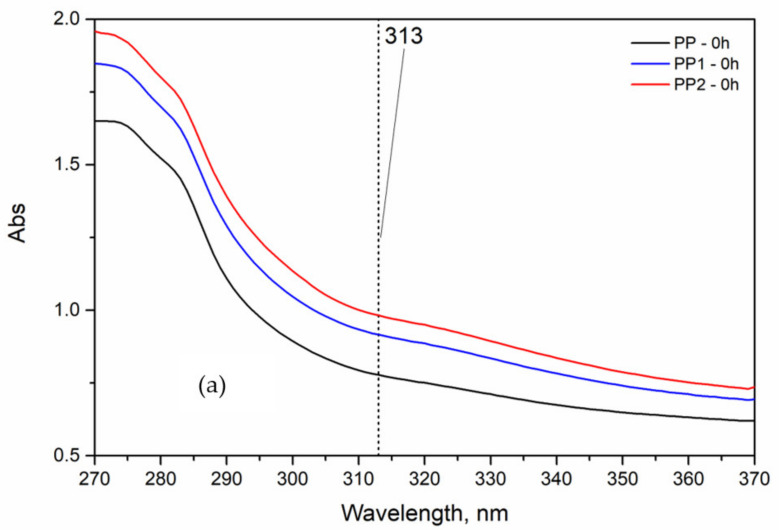

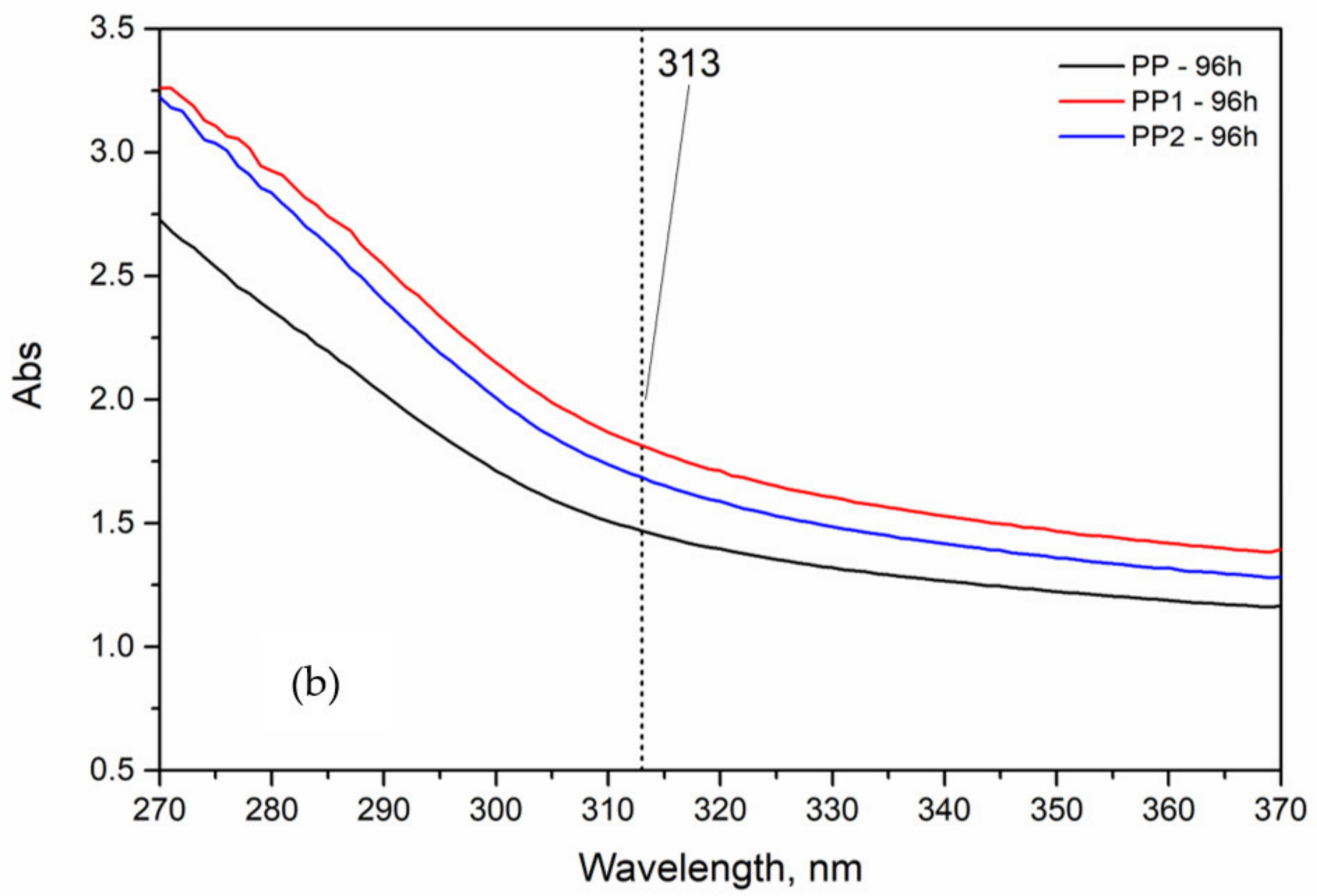
UV absorbance of the polypropylene and polypropylene nanocomposites: (**a**) virgin, (**b**) after 96 h weathering time.

The protective effect can be due to the barrier to the oxygen exerted by the nanoparticles, but also to some absorbance of the UV radiation in the wavelength of the UV lamps (maximum peek at 313 nm). Indeed, in Figure 13 the UV spectra of the two unexposed nanocomposites show a slight absorbance in this wavelength range. That decreases the amount of UV energy acting on the PP macromolecules.

**Figure 14:**
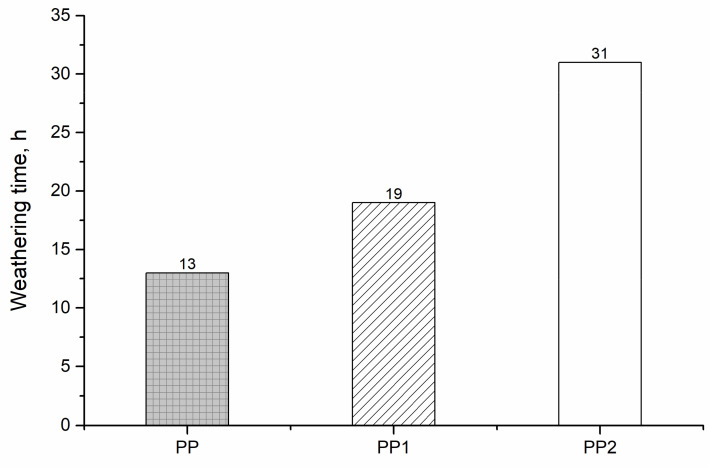
Time at which the elongation at break reaches one half of its initial value (hours) of the polypropylene and polypropylene nanocomposites.

In Figure 14**,** the time at which the elongation at break reached one half of the initial value is reported.

Similar to the mechanical properties, the improvement of this time is relatively low, going from 1 to 2% of nanofiller; nevertheless, this is a doubling of the content of the silica. It is evident that this time increases but less than linearly with the silica content. This behaviour can be attributed to the same effect already discussed for the mechanical properties. The total surface of the silica particles of the average diameter of the nanoparticles. This means that the barrier to the oxygen does not increase linearly with the content of silica.

## 4. Conclusions

The nanocomposite samples investigated in this work show a very interesting behaviour, both in the molten state and in the solid state. In the molten state, indeed, the two nanocomposite samples show flow curves lower than that of the pure matrix. Usually, the presence of inert particles tends to increase the value of the viscosity of the matrix. This behaviour has been interpreted in terms of very scarce adhesion between the two phases. In the solid state, on the contrary, an improvement of the rigidity and of the thermomechanical resistance is observed. The elastic modulus and the tensile strength increase in the presence of the inert silica nanoparticles and, as expected, the elongation at break decreases. The remarkable improvement of the thermomechanical resistance is particularly impressive considering the very small amount of the nanofiller.

The increase in the mechanical properties, however, is not linear with the content of silica. This behaviour has been explained considering the aggregation of nanoparticles in the more loaded sample that increases the particle size and decreases the contact area between the two phases. The decrease in the contact area makes the transmission of the stress from matrix to filler less efficient, reducing the reinforcing action of the filler.

The presence of the silica improves the photostability of the polypropylene matrix due to some barrier to the oxygen exerted by the silica nanoparticles and to some absorbance of the UV radiation by the same nanoparticles similar to the elastic mechanical properties. The protection is not proportional to the content of silica. This behaviour, similar to that of the mechanical properties, has again been interpreted in terms of the dimensions of the nanoparticles that grow with an increasing concentration of silica and a decreasing surface barrier to oxygen.

## Figures and Tables

**Table 1:** Melting temperature, enthalpy of fusion and crystallinity of polypropylene and polypropylene nanocomposites.

	Tm, °C	ΔH_m_, J/g	X_C_, %
PP (100/0 wt/wt%)	176.4	89.4 ± 0.9	43.1
PP1 (99/1 wt/wt%)	177.2	91.8 ± 1.2	44.7
PP2 (98/2 wt/wt%)	177.3	91.1 ± 1.0	44.9

**Table 2:** Melting temperature, enthalpy of fusion and crystallinity of polypropylene and polypropylene nanocomposites after 96 h of weathering time.

	T_m_, °C	ΔH_m_, J/g	X_C_, %	X_C_ (t)/X_C_ (t_0_)
PP (100/0 wt/wt%)	157.3	129.5 ± 1.2	62.5	1.45
PP1 (99/1 wt/wt%)	154.2	130.0 ± 1.0	63.4	1.42
PP2 (98/2 wt/wt%)	154.3	127.6 ± 0.6	62.9	1.40

Tm, ΔHm, X_C_ and X_C_ (t)/X_C_ (t_0_) are the melting temperature, enthalpy of fusion, crystallinity and relative degree of crystallinity, respectively.

## Data Availability

The data presented in this study are available on request from the corresponding author.
